# Supplementary material for: A comprehensive map of the sequence basis of transcription initiation in humans
Source: bioRxiv. 2026 Oct 30:2026.10.29.762743. Preprint. [Version 1] doi: 10.64898/2026.10.29.762743 (PMC13734087; doi:10.64898/2026.10.29.762743)
Supplement: Supplementary file 1 [file NIHPP2026.10.29.762743v1-supplement-2.pdf]

bioRxiv preprint doi: https://doi.org/10.64898/2026.10.29.762743; this version posted October 30, 2026. The copyright holder for this preprint (which was not certified by peer review) is the author/funder, who has granted bioRxiv a license to display the preprint in perpetuity. It is made available under a CC-BY 4.0 International license.

408 # Supplementary Figures

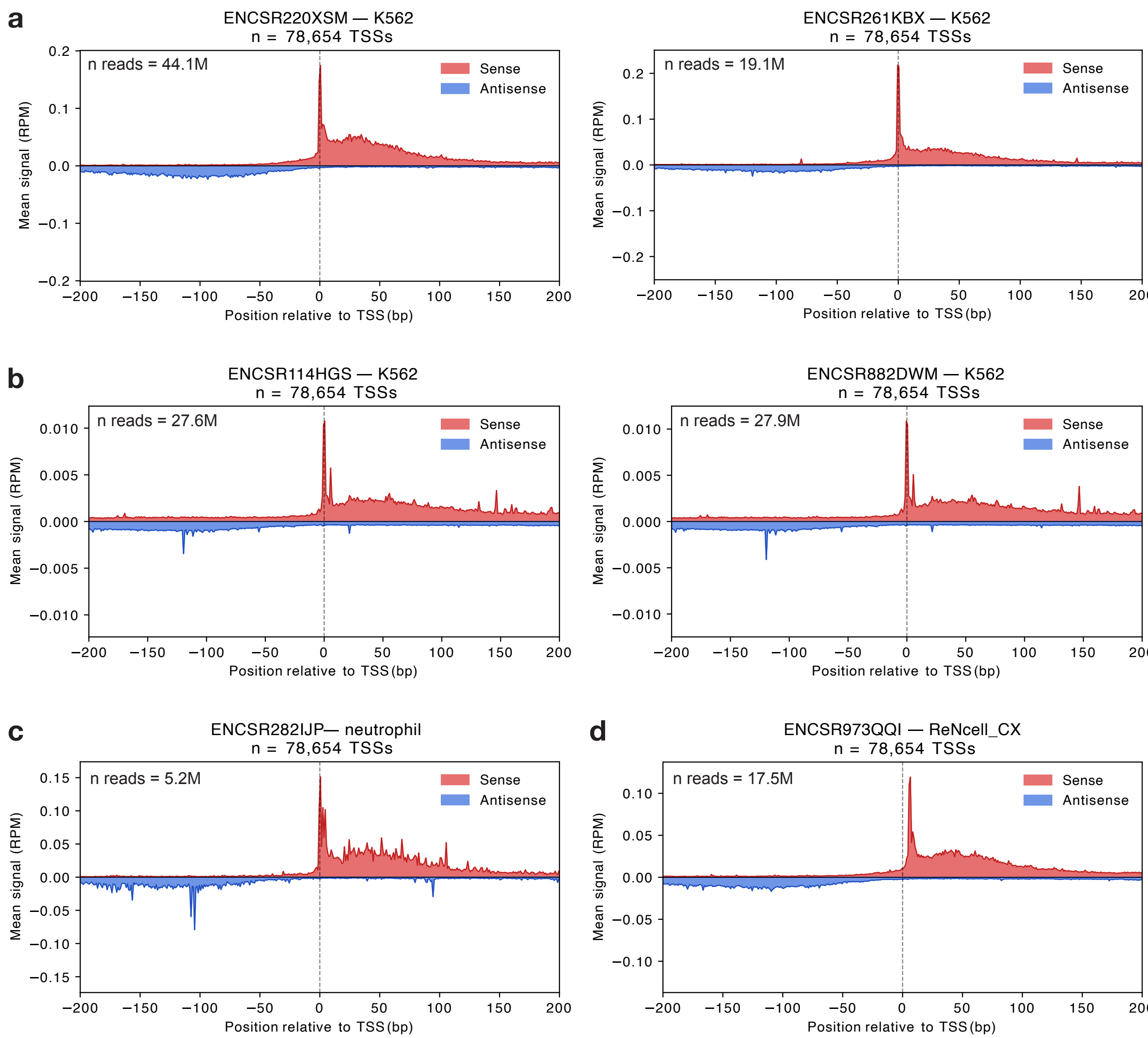


Figure S1: **TSS-centered PRO-cap metaplots illustrating dataset quality-control categories.** Mean strand-oriented PRO-cap signal across 78,654 GENCODE v49 gene transcription start sites on canonical chromosomes, shown from −200 to +200 bp relative to the annotated TSS and normalized to RPM. **a.** Representative K562 experiments judged good (ENCSR220XSM; 44.1 million reads) or acceptable (ENCSR261KBX; 19.1 million reads). **b.** K562 experiments generated with uncapped-RNA enrichment (ENCSR114HGS and ENCSR882DWM), showing strongly reduced TSS enrichment despite read depths of 27.6 and 27.9 million, respectively. **c.** A very low-depth neutrophil experiment (ENCSR282IJP; 5.2 million reads). **d.** The manually excluded ReNcell CX experiment (ENCSR973QQI; 17.5 million reads), showing anomalous downstream displacement of the sense-strand signal.

bioRxiv preprint doi: https://doi.org/10.64898/2026.10.29.762743; this version posted October 30, 2026. The copyright holder for this preprint (which was not certified by peer review) is the author/funder, who has granted bioRxiv a license to display the preprint in perpetuity. It is made available under a CC-BY 4.0 International license.

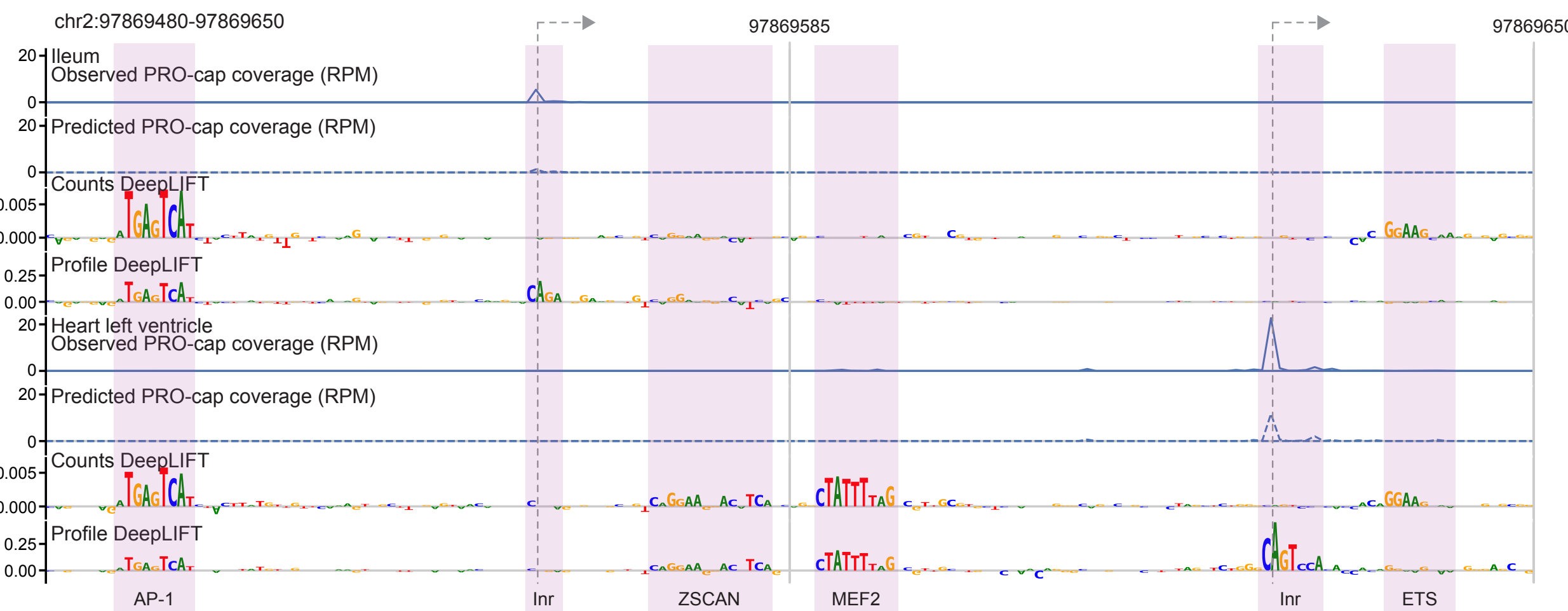


Figure S2: **Models implicate cell-type specific TFs in differential TSS usage across tissues.** Example of an intronic candidate enhancer (ENCODE SCREEN EH38E2017378 [1], within *TMEM131*) in ileum (above) and heart left ventricle (below), showing observed and predicted PRO-cap coverage in RPM, DeepLIFT scores, and manually annotated motif instances. Counts DeepLIFT scores are rescaled to RPM.

bioRxiv preprint doi: https://doi.org/10.64898/2026.10.29.762743; this version posted October 30, 2026. The copyright holder for this preprint (which was not certified by peer review) is the author/funder, who has granted bioRxiv a license to display the preprint in perpetuity. It is made available under a CC-BY 4.0 International license.

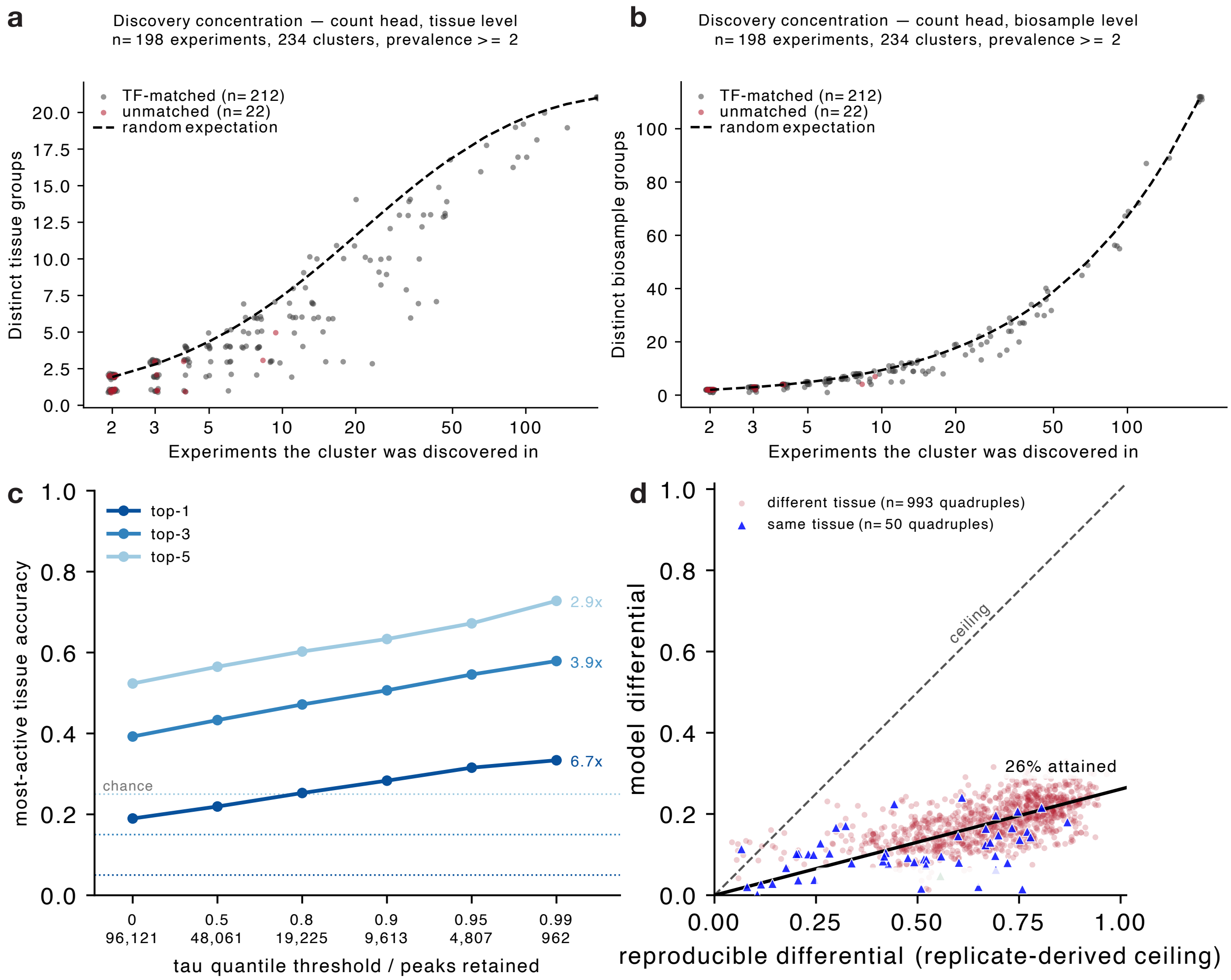


Figure S3: **Robustness of motif lexicon estimates and sequence-based prediction of cross-cell type initiation differences. a.** Discovery concentration on the deduplicated compendium at tissue level (21 groups): distinct tissue groups versus experiment prevalence for each cluster. Points below the dashed expectation curve indicate tissue-concentrated discovery. TF-matched clusters ($n$ = 111) and unmatched clusters ($n$ = 22) shown separately. **b.** Same as **a** at biosample level (112 groups). Points track the expectation curve more closely, confirming that tissue-level concentration reflects lineage restriction across distinct biosamples rather than replicate redundancy from heavily sequenced cell lines. **c.** Top-1, top-3 and top-5 accuracy for identifying the observed most-active tissue among 20 groups across increasing tissue-specificity $\tau$ quantile thresholds. Numbers below the axis indicate peaks retained; dotted lines show chance accuracy. **d.** Predicted differential signal plotted against its replicate-derived ceiling for pairs of replicated biosamples. Only positive ceilings are shown; the dashed line denotes the ceiling and the solid line is a through-origin fit (slope = 0.26).
